# Microfluidic Synthesis of Ca-Alginate Microcapsules for Self-Healing of Bituminous Binder

**DOI:** 10.3390/ma11040630

**Published:** 2018-04-19

**Authors:** Benan Shu, Shaopeng Wu, Lijie Dong, Qing Wang, Quantao Liu

**Affiliations:** 1 State Key Laboratory of Silicate Materials for Architectures, Wuhan University of Technology, Wuhan 430070, China; shuba@whut.edu.cn (B.S.); liuqt@whut.edu.cn (Q.L.); 2 State Key Laboratory of Advanced Technology for Materials Synthesis and Processing, School of Materials Science and Engineering, Wuhan University of Technology, Wuhan 430070, China; lijie@whut.edu.cn (L.D.); 254546@whut.edu.cn (Q.W.)

**Keywords:** Ca-alginate microcapsules, microfluidic, self-healing, bitumen

## Abstract

This work aims to develop an original alginate micro-emulsion combining with droplets microfluidic method to produce multinuclear Ca-alginate microcapsules containing rejuvenator for the self-healing of bituminous binder. The sizes of the Ca-alginate microcapsules could be easily controlled by tuning flow rates of the continuous and dispersed phases. The addition of a surfactant Tween80 not only improved the stability of the emulsion, but it also effectively reduced the size of the microcapsules. Size predictive mathematical model of the microcapsules was proposed through the analysis of fluid force. Optical microscope and remote Fourier infrared test confirmed the multinuclear structure of Ca-alginate microcapsules. Thermogravimetric analysis showed that the microcapsules coated with nearly 40% rejuvenator and they remained intact during the preparation of bitumen specimen at 135 °C. Micro self-healing process of bituminous binder with multinuclear Ca-alginate microcapsules containing rejuvenator was monitored and showed enhanced self-healing performance. Tensile stress-recovery test revealed that the recovery rate increased by 32.08% (in the case of 5% microcapsules), which meant that the Ca-alginate microcapsules containing rejuvenator could effectively enhance the self-healing property of bituminous binder.

## 1. Introduction

Self-healing materials as a kind of intelligent material have attracted increasing interest because it can automatically repair the internal structural damage of the material [1,2,3,4,5,6]. Bitumen-a is widely used material in pavements has a limited service life. The bituminous pavement would produce micro cracks under the action of load, temperature, ultraviolet light, and so on. Although it has a certain self-healing ability, it will develop into a macro crack under the action of continuous traffic load [7,8,9,10]. In view of this, bitumen containing various capsules with self-healing property to prolong its service life has been widely investigated [11,12,13,14,15]. However, synthesis of those capsules generally has some disadvantages: limited encapsulation, high cost, and complex synthesis process. Sodium alginate, for its low cost, ease of use, and non-toxicity has been intended to be used to prepare microcapsules for the self-healing of bitumen. Garcia et al. [16,17] reported on the fabrication of porous calcium alginate spheres containing sunflower oil for the self-healing of bitumen mixture. The oil content of the capsules prepared by this method was limited and the particle size was too large to repair the micro cracks in the bitumen. Size distribution would influence self-healing performance of microcapsules in bitumen [18,19]. In view of this, different-sized and high rejuvenator loading calcium alginate microcapsules are badly needed.

At present, there are two mechanisms for the synthesis of alginate microcapsules: forward gelation and reverse gelation. For the forward gelation, Ren et al. [20] reported using oil/water/oil (O/W/O) double emulsions as templates to synthesis alginate microcapsules with oil core by a microfluidic device. The effect of the inner and outer diameters of quartz glass tube on the size of Ca-alginate microcapsules was analyzed. Kuan-Wen Yeh et al. [21] reported that Ca-alginate microcapsules with tea-tree oil were synthesized after the addition of calcium chloride solution to the sodium alginate solution in the flask with mixing and the release model was studied. The microcapsules had a large particle size distribution. Romanowsky et al. [22] have demonstrated a parallelized microfluidic design by making double emulsions of water/octanol/water (W/O/W) to prepare various encapsulation like Ca-alginate microcapsules with oil cores. Supaporn et al. [23] synthesized the Ca-alginate microcapsules containing eucalyptus oil with a narrow size distribution via Shirasu porous glass (SPG) membrane. The concentration of alginate solution, temperature, and release model were investigated. Chew et al. [24] proposed that Kenaf seed oil was encapsulated using co-extrusion technology with a high methoxyl pectin (HMP)-enhanced alginate shell. Schmit et al. [25] showed that the oil droplets were pumped into alginate/CaCO_3_ solution and the formed O/W emulsion was added drop wise to the oil–acetic acid bath through a co-flow junction microfluidic device. However, the distribution and dissolution of CaCO_3_ limited the morphology of the capsules. Marquis et al. [26] described two-step approach allowing the encapsulation of several oil micro-droplets within alginate micro-gels. Cotton cellulose nanocrystals and calcium carbonate was used to prepare Pickering emulsion in the first step, oil micro-droplets resulting from the Pickering emulsion were encapsulated within alginate micro-gels using microfluidics. Evandro Martins et al. [27,28] proposed a technique of oil encapsulation in Ca-alginate microcapsules by inverse gelation. The oil containing calcium ions dropped into the sodium alginate solution, and the calcium ions diffused from inside to outside to obtain capsules. The reverse gelation mechanism has also been extensively studied by other researchers [29,30,31,32]. However, those methods usually either involved multiple fluids to be controlled simultaneously in the process of experiments, or cumbersome preparation which makes mass production difficult. Furthermore, the diffusion of calcium ions makes it easy for alginate solution to form a whole block of hydrogel and the secondary dissolution of capsules in reverse gelation makes it difficult to produce separable capsules. Moreover, the formation mechanism of droplets in microfluidic devices has not been deeply studied.

To the best of our knowledge, it seems there is no published paper concerning the synthesis of using alginate micro-emulsion combining droplets microfluidic. In view of this, this paper performed a micro-emulsion of alginate and rejuvenator combining with droplets microfluidic method to produce Ca-alginate microcapsules. The size can be easily controlled and the formation mechanism of the emulsion droplets in microfluidic device was deeply studied from the point of view of the fluid force. Then a size predictive mathematical model of the microcapsules was proposed. In addition, the self-healing performance of bitumen containing the microcapsules was investigated. This research provides possibilities for industrial large-scale production of Ca-alginate microcapsules containing rejuvenators, extending the service life of bituminous materials—such as bitumen highways, bitumen waterproofing layers, and bitumen anticorrosive materials—and realizing development of a more sustainable and green construction industry related to bitumen materials.

## 2. Materials and Methods

### 2.1. Materials

Sodium alginate, Tween80, and anhydrous calcium chloride (CaCl_2_·2H_2_O) were purchased from Sinopharm Chemical Reagent Co., Ltd. (Beijing, China). 70A (70A is an abbreviation of bitumen with 60/80 pen grade) bitumen was supplied by KOCH Bitumen Co., Ltd. (Wuhan, China) and properties are shown in Table 1. Rejuvenator was obtained from Hubei Bo Run Chemical Technology Co., Ltd. (Wuhan, China) and its properties are shown in Table 2. Oil was purchased from Jiali grain and Oil Co., Ltd. (Beijing, China). All of the reagents were used without further purification.

### 2.2. Preparation of O/W Emulsions

300 mL deionized water, 3 g alginate,100 mL rejuvenator, and different volume fractions of Tween80 (0–2.0%) were mixed and stirred for 10 min at 50 °C. Then the mixture was sheared for 15 min with a high-speed shearing mixer running at 8000 rpm.

### 2.3. Fabrication of Ca-Alginate Microcapsules by Microfluidic

**Figure 1:**
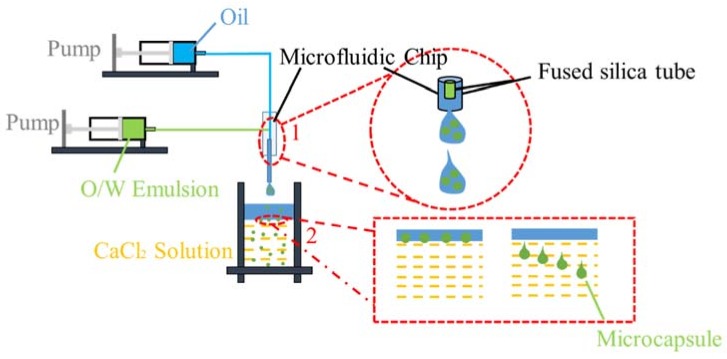
Schematic showing the droplets microfluidic device with a co-axial flow focusing geometry.

A microfluidic device with a co-axial flow focusing geometry was designed as Figure 1. For the outer phase, oil was pumped (NE-1000, ERA Syringe Pump, New Era Pump systems Inc, Washington, WA, USA) through a fused silica capillary tube (interior diameter (ID) 0.8 mm and outside diameter (OD) 1.0 mm) with a rate of changing from 3 mL·h^−1^ to 9 mL·h^−1^. The inner phase, emulsion was pumped through a fused silica capillary tube (interior diameter (ID) 400 μm and outside diameter (OD) 600 μm) with a rate of changing from 3 mL·h^−1^ to 9 mL·h^−1^. Emulsion droplets with different diameters can be generated at the different flow rate of continuous phase and dispersed phase owing to the effect of fluid force. When the droplets were immersed in calcium chloride solution, the substitution of calcium ions to sodium ions causes the crosslinking of alginate ions into membrane, and finally microcapsules with different diameters can be synthesized. The microcapsules were retained in calcium chloride solution for 4 h, and the calcium chloride solution containing microcapsules was filtered and the Ca-alginate microcapsules with rejuvenator was left in oven at 110 °C for 12 h. The oil on the surface of calcium chloride solution could be repeatedly recycled by a separating liquid funnel.

### 2.4. Characterization of Ca-Alginate Microcapsules

Polarizing microscope (ECLIPSE LV100N POL, Nikon, Tokyo, Japan) and fluorescence microscope (CX23, OLYMPUS, Wuhan, China) were used to image microcapsules.

A thermal analysis test with a working temperature range of 50–600 °C was conducted. TGA/DSC simultaneous thermal analyzer (STA449c/3/G, Berlin, Germany ) was used in this paper. The heating rate was controlled at 10 °C/min with a maximum temperature of 600 °C. At the same time, high-purity nitrogen ambient gas was applied at a flow rate of 500 mL/min. During the pyrolysis process, the organic volatile substances of the polymers were decomposed to low molecular weight products. The relationship between the mass of the test sample and the temperature can be obtained from the TGA/DSC tests, which can be used to analysis the rejuvenator content in the microcapsules as well as the thermal stability of the microcapsules.

The FT-IR test can detect functional groups in a material which can be used to determine whether a chemical reaction occurs by comparing the differences in functional groups. The FT-IR test was conducted through an infrared spectrum instrument (Nexus, Thermo Nicolet Corporation, Washington, WA, USA) to confirm the successful synthesis of the microcapsule with wavelengths ranging from 400 cm^−1^ to 4000 cm^−1^.

### 2.5. Self-Healing Evaluation of Bitumen Containing Ca-Alginate Microcapsules

70# bitumen was aged for 5 h at 163 °C according to the standard JTG E20-2011 [33]. After that, different contents (i.e., by weight) Ca-alginate microcapsules was added slowly to the aged bitumen at 135 °C with a stirring speed of 200 rpm. Then the mixture was poured into the strips of 50 mm length, 10 mm width, and 2 mm height. These bitumen strips were kept at 0 °C for 1 h, and then fractures (approximately 20 μm) was made using a blade. Finally, the bitumen strips with fracture were transferred to the glass slide. Fracture morphology of samples with different content Ca-alginate microcapsules and self-healing time was observed by optical microscopy and fluorescence microscopy as well as tensile stress-recovery test to evaluate the self-healing property of bitumen containing Ca-alginate microcapsules.

## 3. Results and Discussion

### 3.1. Synthesis Mechanism of the Microcapsules

**Figure 2:**
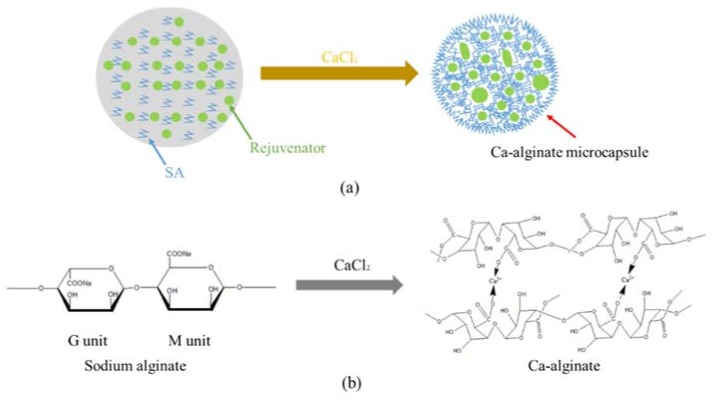
Schematic for the formation of Ca-alginate microcapsules with rejuvenator core (**a**) and crosslinking mechanism of Ca-alginate (**b**).

Micro-emulsion of rejuvenator in sodium alginate solution (O/W) was formed with high-speed shearing. The synthesis mechanism of Ca-alginate microcapsules containing rejuvenator is shown in Figure 2. Schematic in Figure 2a shows the formation of Ca-alginate microcapsules with rejuvenator core. Once in contact with the calcium chloride solution, the alginate ions on the surface of the droplets would cross-link to form a membrane under the substitution of sodium ions by calcium ions. As the reaction goes on, calcium ions continue to diffuse and invade the membrane because of the influence of calcium ions concentration difference between the inside and outside of the membrane. Thus micro-droplets of rejuvenator were in situ immobilized in the microcapsules. Some droplets will amalgamate to form large cores due to instability. Finally, multinuclear Ca-alginate microcapsules containing rejuvenator were successfully synthesized. The crosslinking mechanism of Ca-alginate is showed in Figure 2b.

**Figure 3:**
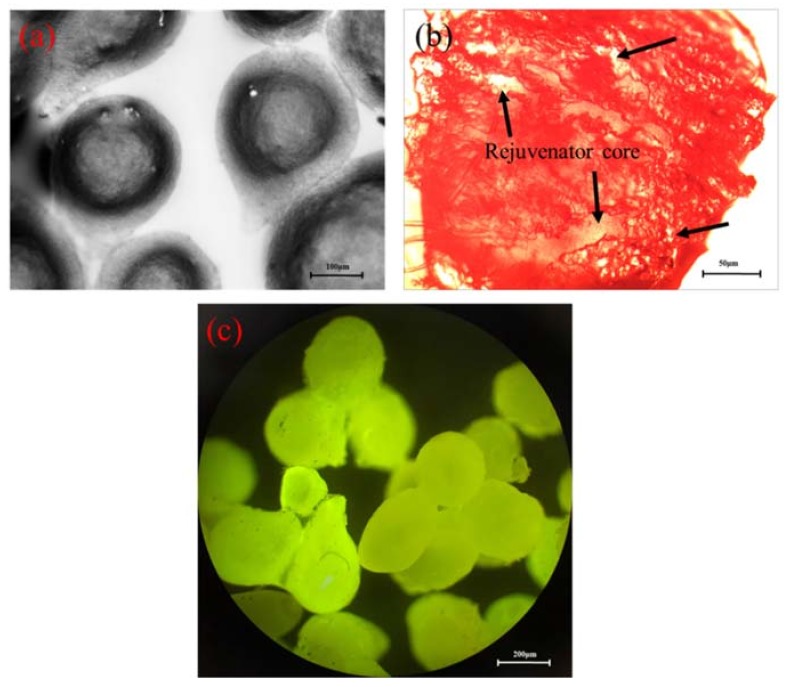
Morphology of the microcapsules ($\vartheta_{o}$: 3 mL·h^−1^, $\vartheta_{e}$ : 5 mL·h^−1^): (**a**,**b**) by optical microscope; (**c**) by fluorescence microscope.

Figure 3a shows the morphology of Ca-alginate microcapsules. The formation of the tail is a result of the forces that droplets subjected at the oil–water interface. When the droplets fall to the oil–water interface, they are subjected to gravity and a viscous force. One part of droplets in calcium chloride solution has formed membrane, but the other part was also emulsion. There was a central extrusion effect on this part generated by viscous force of the rejuvenator phase. It was deformed and form a tail during the drop of emulsion in the case of calcium ions. Figure 3b shows the fracture morphology of Ca-alginate microcapsules. It can be seen that there are many bright spots in the microcapsule center (the area black arrows pointed), which is the core of rejuvenator. Figure 3c shows the fluorescence morphology of microcapsules. The whole microcapsules have a strong fluorescence effect, which is attributed to the rejuvenator with fluorescence effect.

**Figure 4:**
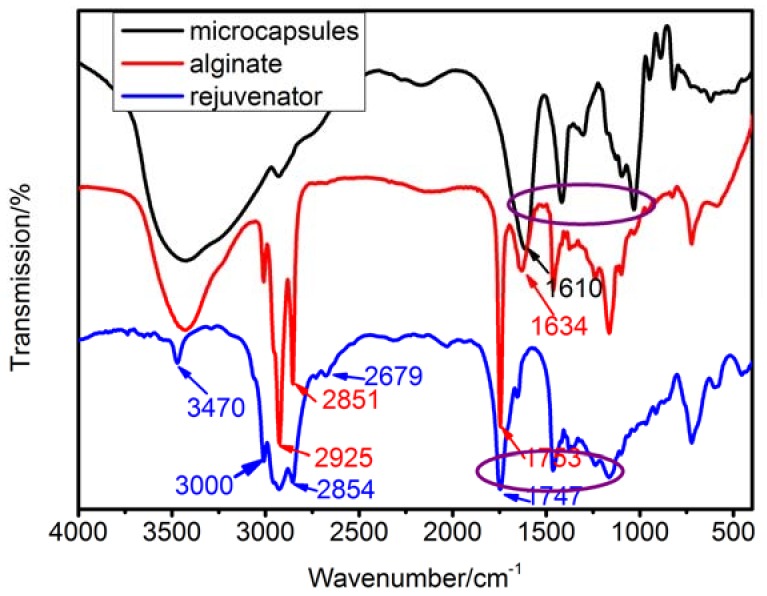
FT-IR test of microcapsules, alginate, and rejuvenator.

The successful synthesis of microcapsules rejuvenator core can be further confirmed by FT-IR test. From the comparison of alginate and microcapsules in Figure 4, it can be seen that 2851 cm^−1^, 2925 cm^−1^ is symmetric telescopic vibration and asymmetric expansion vibration of$\;–{\mathrm{CH}}_{2}–\;$. 1634 cm^−1^ is the telescopic vibration of carboxyl group. These absorption peaks did not appear in the test of microcapsules. The infrared absorption peak from 1747 cm^−1^ to 1161 cm^−1^ moved to the lower wave number, and the intensity of all the absorption peaks became wider and weaker. This result is attributed to the addition of calcium ions made the carboxyl group into crosslinking. G began to fold the accumulation of seaweed ions, changing from the neat and orderly band structure to the curly cross-linking structure, eventually forming a three-dimensional network structure. Due to such steric effect, the intensity of all absorption peaks decreased and some absorption peaks moved to the lower wave number while some other peaks disappeared. For instance, comparison of the infrared spectra of rejuvenator and microcapsules shows that the peak 3470, 3000, 2854, and 2679 cm^−1^ in rejuvenator disappeared after the synthesis of microcapsules; the infrared absorption peak from 1747 cm^−1^ to 1161 cm^−1^ moved to the lower wave number. These changes are because the calcium alginate membrane restricted the spatial displacement of the group in the rejuvenator and made the partial absorption peak disappeared, another partial absorption peak moved to the lower wave number, which indicated that the rejuvenator was coated in Ca-alginate shell. 

### 3.2. Effect of Surfactant on the Size of the Microcapsules

**Figure 5:**
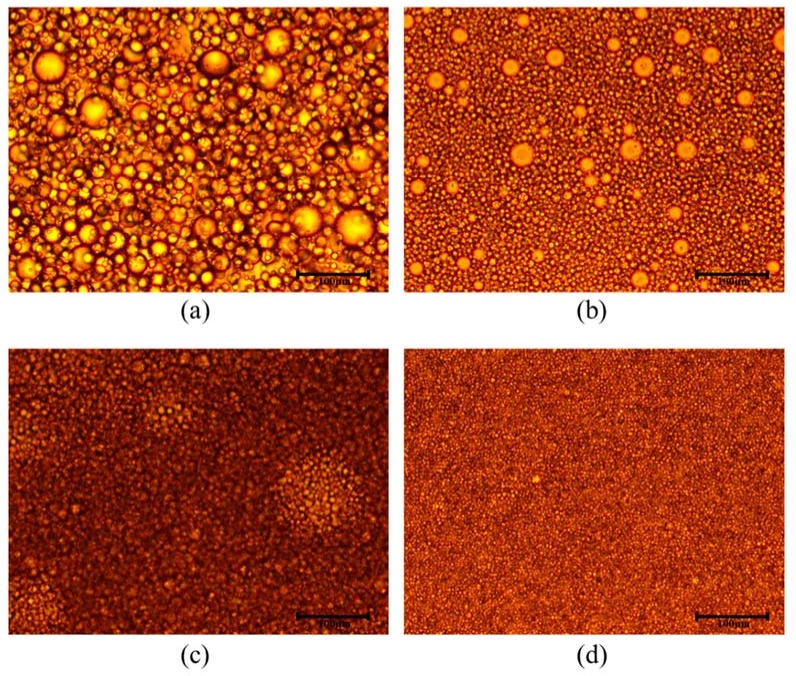
Optical microscopic image of emulsion with different content Tween80: (**a**) 0%; (**b**) 0.5%; (**c**) 1.0%; (**d**) 2.0%.

**Figure 6:**
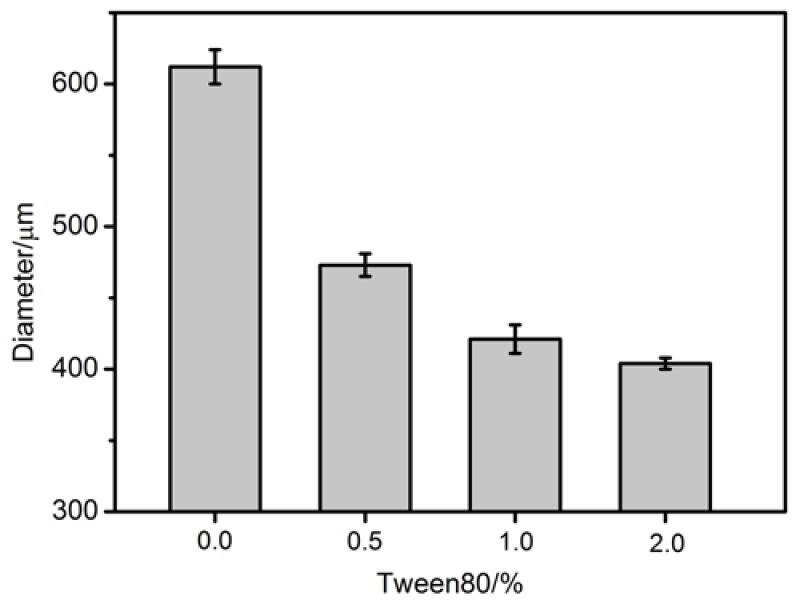
Size of microcapsules synthesized with different content Tween80 ($\vartheta_{o}$: 3 mL·h^−1^, $\vartheta_{e}$ : 5 mL·h^−1^).

During the synthesis of the microcapsules by microfluidic droplets, there is a long retention time of micro-emulsion in the syringe. It is necessary to prepare highly stable emulsion to prevent its stratification in the syringe. The comparison of the micro-emulsion with different content Tween80 was conducted. Figure 5 shows the stability of micro-emulsion at room temperature. It can be seen that the emulsion without Tween80 showed obvious inhomogeneity. For example, there was a large variation in the size of emulsion and the emulsified droplets were unstable and they ruptured quickly. When 0.5% Tween80 was added, the size of emulsion had a significant decrease and there was only a few of the droplets coalesced. The size of emulsion with 1.0% Tween80 was further decreased and the stability was substantially improved and there was no obvious aggregation and fusion. When 2.0% Tween80 was added, the size of emulsion decreased and there was no significant change in stability as compared to the emulsion with 1.0 wt % of Tween80. In view of this, the content of 1.0% emulsion in this study was used. It also showed that the addition of Tween80 significantly reduced the size of the microcapsules (Figure 6). When 0.0, 0.5, 1.0, and 2.0% Tween80 was added, the size of microcapsules ($\vartheta_{o}$: 3 mL·h^−1^, $\vartheta_{e}$ : 5 mL·h^−1^) were about 610, 470, 420, and 400 μm respectively. The addition of surfactant like Tween80 can diminish the interfacial tension of the liquid [34,35,36], which affected the synthesis of Ca-alginate from two different ways. One is that water molecules can be fixed on the surface of the rejuvenator droplets by Tween80 to prevent the aggregation and fusion of the emulsion droplets in alginate solution, thus improving the stability of the micro-emulsion. Another is that the smaller interfacial force makes the adhesive force between the emulsion and the outer surface of the quartz tube decreased. When the velocity of the continuous phase is not changed, the emulsion droplets are more easily formed which means that a smaller shear force generated by the continuous phase is needed to form the same size droplets after surfactant Tween80 is added. Thus, the size of alginate microcapsules decreased after adding Tween80.

### 3.3. Effect of the Flow Rates of Continuous and Dispersed Phases on Microcapsules’ Size

The control of morphology of the microcapsules was performed by tuning the flow rate of continuous (oil) and dispersed (O/W emulsion) phases. Table 3 shows the relation between the size of the microcapsules and the velocity of continuous and dispersed phase. It can be seen that when the flow rate of continuous and dispersed phase was changed between 3 mL·h^−1^ and 9 mL·h^−1^, the microcapsules with diameter from 139 ± 8 μm to 482 ± 16 μm were synthesized. It also revealed that by keeping constant flow rate of dispersed phases, the diameter of the microcapsules decreased with the increase of flow rate of the continuous phase. On contrary, using a constant flow rate for continuous phase, the diameter of the microcapsules increased with the increase of flow rate of the dispersed phase.

### 3.4. Size Predictive Mathematical Model of the Microcapsules

The results can be explained through the interaction of the forces acting on the micro-emulsion droplets. In this experimental method, the formation and morphology of droplets was affected by the four different forces acting on the droplets. They are shear force ($F_{s}$) produced by continuous phase, interfacial force between dispersed phase and outer surface of quartz glass tube ($F_{i}$), thrust produced by the pump ($F_{t}$), and buoyancy force ($F_{b}$) produced by density difference between continuous and dispersed phase.

(1) $$F_{s}=3\pi \times \mu_{o}\times \left(\vartheta_{o}-\vartheta_{e}\right)\times r_{e}$$

(2) $$F_{i}=2\pi \times \gamma \times \sigma_{e}$$

(3) $$F_{t}=\rho_{e}\times \emptyset_{e}\times \vartheta_{e}$$

(4) $$F_{b}=\pi \times \left(\rho_{e}-\rho_{o}\right)\times g\times r^{3}/6$$

$\mu_{o}$ represents viscosity of the continuous phase (oil), $\vartheta_{o}$ and $\vartheta_{e}$ are velocity of the continuous phase and dispersed phase, $r_{e}$ means radius of emulsion droplets, γ is the wetting radius of the emulsion droplet at the inner quartz capillary tube, $\sigma_{e}$ stand for the interfacial tension, $\emptyset_{e}$ stands for flux of the emulsion, $\rho_{e}$ and $\rho_{o}$ are density of the emulsion and oil phase respectively. Bond number ($B_{o}$) was used to evaluate the relationship between buoyancy force and interfacial force

(5) $$B_{o}=\;\Delta \rho gL^{2}/\sigma$$

$\rho_{o}=0.9318$, $\rho_{e}\;$= 0.9832. $B_{o}\ll 1$, so $F_{b}$ could be ignored [37]. Replace the Formulas (1)–(3) into Formula (5)

(6) $$F_{i}=F_{t}+F_{s}$$

(7) $$r_{e}=\left[2\pi \times \gamma \times \sigma_{e}+\vartheta_{e}\times \left(2\pi \mu_{o}-\rho_{e}\times \emptyset_{e}\right)\right]/3\pi \times \mu_{o}\times \vartheta_{o}.$$

**Figure 7:**
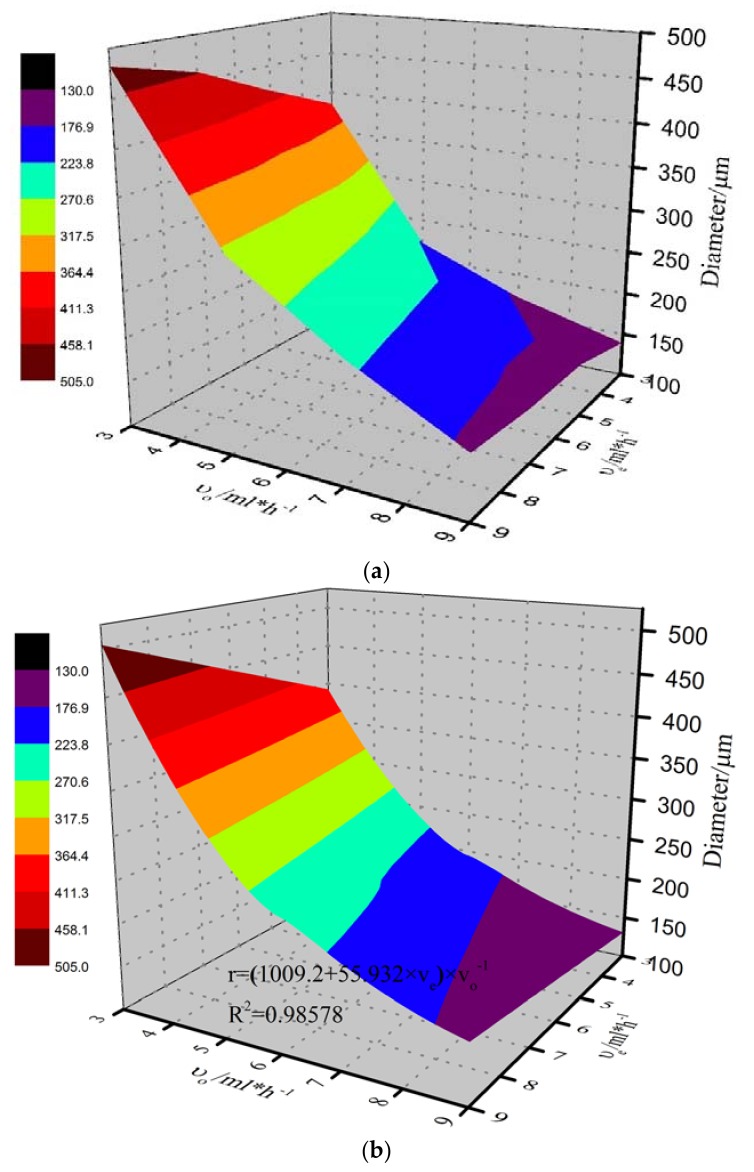
Three-dimensional experimental image (**a**) and fitting image (**b**) of diameter of the microcapsules versus flow rate of continuous phase (oil) and dispersed phase (alginate emulsion).

γ, $\sigma_{e}$, $\mu_{o}$, $\rho_{e}$, and $\emptyset_{e}$ are determined by the physical properties of the micro-emulsion and microfluidic device. So it can be drawn that $r_{e}\propto \vartheta_{e}$, $r_{e}\propto 1/\vartheta_{o}$. When surfactant Tween80 was added, interfacial force of the emulsion was decreased namely $\sigma_{e}$ decreased, thus r decreased when $\vartheta_{e}$ and $\vartheta_{o}$ were constant. The experimental results conform to the law of the formula deduced. A three-dimensional experimental image of diameter of the microcapsules versus flow rate (3 mL·h^−1^–9 mL·h^−1^) of continuous phase and dispersed phase is shown in Figure 7a and the fitting image is shown in Figure 7b. A fitting formula was proposed.

(8) $$r_{e}=(1009.2+55.932\times \vartheta_{e})\times \vartheta_{o}{}^{\left(-1\right)}\times R^{2}=0.98578$$

### 3.5. Self-Healing Property of Bitumen with Multinuclear Ca-Alginate Microcapsules

**Figure 8:**
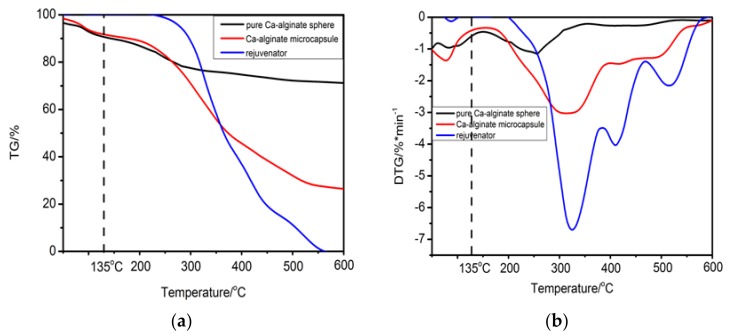
Thermal analysis of alginate, Ca-alginate microcapsules and rejuvenator: (**a**): TG vs temperature, (**b**): DTG vs. temperature.

The effect of Ca-alginate microcapsules on the self-healing performance of bituminous binder was studied for the first time. The thermal stability of the microcapsules was investigated and showed in Figure 8. The thermogravimetry test in Figure 8a shows that thermogravimetric curves of the pure Ca-alginate sphere and microcapsules had similar characteristics. Nearly 40% rejuvenator was coated in Ca-alginate microcapsules. It also can be seen from Figure 8b that the initial decomposition temperature of the rejuvenator was 200 °C, the maximum decomposition temperature was 325 °C. The microcapsules and Ca-alginate sphere had slight mass loss at 100 °C, which was the evaporation of water. The maximum decomposition temperature of the microcapsules was 313.5 °C, and the maximum decomposition temperature of the Ca-alginate sphere was 256 °C which reveal that, when a bitumen specimen with multinuclear Ca-alginate microcapsules containing rejuvenator is prepared at 135 °C, the microcapsules probably keep intact.

#### 3.5.1. Micro Self-Healing Process of Bituminous Binder with Microcapsules

**Figure 9:**
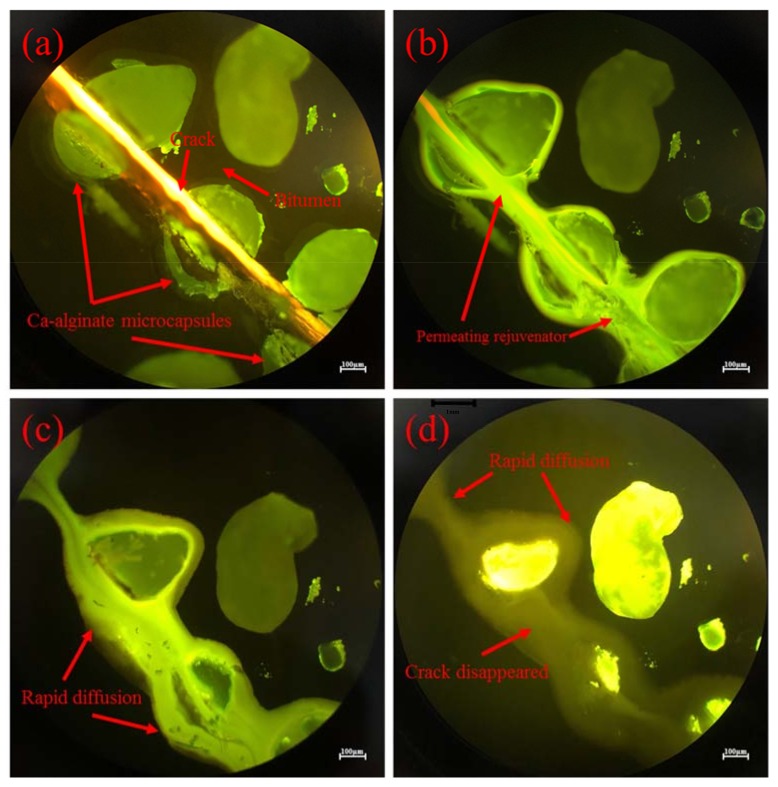
Micro self-healing process of bituminous binder with Ca-alginate microcapsules characterized by fluorescence microscope and optical microscope test (at 20 °C): (**a**) once a crack was produced; (**b**) after 30 s; (**c**) after 1 min; (**d**) after 4 h.

The self-healing mechanism of microcapsules for bitumen is that when the crack develops to meet microcapsules, the shell materials of microcapsules will be ruptured because of stress concentration at the tip of a crack. Then rejuvenator release and flow into the crack owing to capillary action. Rejuvenator will dissolve bitumen on both side of the crack. Then bituminous molecules spread quickly to the crack center under the effect of Brownian movement and concentration difference. In the case of the π-stacking of the aromatic rings and amphoteric character of aromatic molecules, the rejuvenator with high proportion of aromatic components can improve adhesion between bituminous molecules. Thus, the self-healing property of bitumen can be enhanced [38,39,40]. Micro self-healing process of bituminous binder containing multinuclear Ca-alginate microcapsules containing rejuvenator was monitored by optical microscope and fluorescence microscope and showed in Figure 6. A crack with a width of nearly 20 μm was produced in Figure 9a. Microcapsules were ruptured on the fracture surface of bitumen. After 30 s, it can be seen from Figure 9b that massive rejuvenator with intense fluorescence effect released and flowed into crack under the effect of capillary action. Part of the rejuvenator diffused into bituminous binder area surrounding those microcapsules. After 1 min, from Figure 9c, it can be seen that rejuvenator in the crack began to rapidly diffuse to the both sides of the crack. The asphalt on both sides of the crack was partly dissolved and diffused to the crack center rapidly because of concentration difference. After 4 h, it can be seen from Figure 9d that the crack disappeared. The microcapsules were partially embedded in the low intensity area. According to the self-healing mechanism, low intensity fluorescence area (in Figure 9d) is the mixture of bitumen and rejuvenator, which was attributed to Brownian motion of bituminous molecules and rejuvenator molecules.

#### 3.5.2. Tensile Stress-Recovery Test of Bituminous Binder with Microcapsules

**Figure 10:**
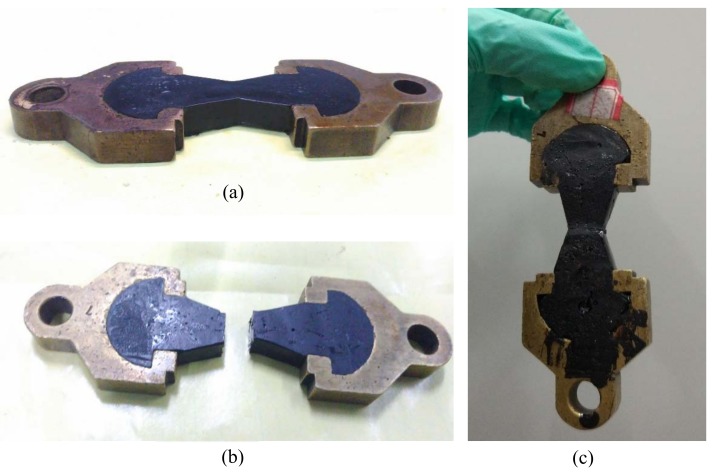
The process of tensile stress-recovery test: (**a**) ductility specimen at low temperature; (**b**) brittle fracture of specimen; (**c**) the two parts were connected again at low temperature.

Tensile stress-recovery test was conducted to evaluate self-healing property of bitumen containing Ca-alginate microcapsules (Figure 10). Bitumen mixing the microcapsules was poured in to the mold at 135 °C (Figure 10a). Then the specimen was kept for one hour at −10 °C. After that the specimen was snapped and the tensile stress was recorded (Figure 10b). Further, the two parts were contacted again and kept flat for one day at low temperature (10 °C). Finally, it can be seen from Figure 10c that the two parts were reconnected to a complete specimen, and the tensile stress was partially restored. The entire process was carried out under low temperature conditions because it guaranteed the brittle fracture of bitumen, and at the same time, the self-healing effect of rejuvenator on bitumen was better exhibited, and the effect of the self-repairing ability of the bitumen itself on the experimental results at low temperature could be minimized.

**Figure 11:**
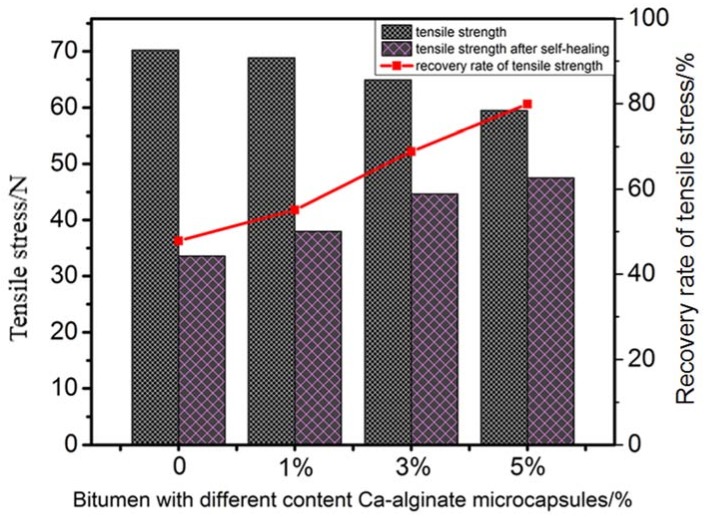
Tensile stress and recovery rate of bitumen with different content Ca-alginate microcapsules.

The self-healing property of bituminous binder with multinuclear Ca-alginate microcapsules containing rejuvenator was evaluated by recovery rate of tensile stress and result was showed in Table 4 and Figure 11. It revealed that initial tensile stress of bitumen had a slight decrease, namely from 70.23 N to 61.48 N, with increase of the content of Ca-alginate microcapsules. The location of the microcapsules in bitumen structure is like a defect. With the increase of the content of Ca-alginate microcapsules, the defects in the bitumen structure also increased, which led to the decrease of mechanical properties of the bitumen specimens. After the self-healing time, the tensile stress of those samples got a different degree of recovery. Tensile stress increased from 33.58 N to 49.12 N with the addition of microcapsules. Furthermore, with the increase of the microcapsules content from 0% to 5%, the recovery rate of tensile stress was increased from 47.81% to 79.89%, which meant that the self-healing property of bitumen with multinuclear Ca-alginate microcapsules containing rejuvenator was enhanced.

## 4. Conclusions

In this paper, we proposed a microfluidic method to synthesize multinuclear Ca-alginate microcapsules containing rejuvenator. The effect of surfactant and flowrate on the emulsion and the size of microcapsules was studied. Further, the self-healing properties of bituminous binder with those microcapsules was investigated. The following conclusions can be drawn:

1. Multinuclear Ca-alginate microcapsules containing rejuvenator were successfully synthesized by a microfluidic droplet device.
2. The microcapsules with size ranging from 139 μm to 482 μm has be synthesized. The addition of surfactant Tween80 could effectively improve stability of the emulsion and reduce the size of the microcapsules. The size could be easily controlled by changing the flow rates of continuous phase and dispersed phase.
3. The formation mechanism of the emulsion droplets in the microfluidic device was explained by force analysis of the droplets, and the prediction model of the microcapsules size was thus obtained.
4. Micro self-healing process of bituminous binder with those microcapsules was monitored and showed the enhanced self-healing performance. Tensile stress-recovery test revealed that the recovery rate increased by 32.08% (in the case of 5% microcapsules), which meant that the multinuclear Ca-alginate microcapsules containing rejuvenator we fabricated could effectively enhance the self-healing property of bituminous binder.

## Figures and Tables

**Table 1:** Properties of the 70A bitumen binder.

Bitumen	Penetration/0.1 mm (20 °C)	Softening Point/°C	Ductility (15 °C)
70A	68.7	48.5	>100 cm

**Table 2:** Properties of the rejuvenator.

Apparent Viscosity/Pa s (60 °C)	Flash Point/°C	Saturates/%	Aromatics/%	Density/g·cm^−3^ (15 °C)
0.285	240	21.07	67.4	0.935

**Table 3:** Diameter of microcapsules versus flowrate of continuous (3 mL·h^−1^–9 mL·h^−1^) and dispersed phases (3 mL·h^−1^–9 ml·h^−1^).

$\vartheta_{e}$/mL·h^−1^	$\vartheta_{o}$/mL·h^−1^			
	**3**	**5**	**7**	**9**
3	385 ± 4 μm	221 ± 11 μm	172 ± 5 μm	139 ± 8 μm
5	421 ± 12 μm	272 ± 8 μm	203 ± 3 μm	159 ± 8 μm
7	460 ± 8 μm	290 ± 5 μm	218 ± 9 μm	161 ± 13 μm
9	482 ± 16 μm	311 ± 3 μm	232 ± 11 μm	169 ± 5 μm

**Table 4:** Tensile stress of bitumen specimens with different content Ca-alginate microcapsules.

Specimen	Tensile Stress (N)	Tensile Stress after Recovery (N)	Strength Recovery Rate (%)
0	70.23	33.58	47.81
1%	68.85	37.96	55.13
3%	65.93	44.68	68.81
5%	61.48	49.12	79.89
